# Aging research using the common marmoset: Focus on aging interventions

**DOI:** 10.3233/nha-180046

**Published:** 2019-09-24

**Authors:** Corinna N. Ross, Adam B. Salmon

**Affiliations:** a Department of Science and Mathematics, Texas A&M University San Antonio, San Antonio, TX, USA; b The Sam and Ann Barshop Institute for Longevity and Aging Studies and The University of Texas Health Science Center at San Antonio, San Antonio TX, USA; c Department of Molecular Medicine, The University of Texas Health Science Center at San Antonio, San Antonio, TX, USA; d Geriatric Research, Education and Clinical Center, South Texas Veterans Health Care System, San Antonio, TX, USA

**Keywords:** Marmoset, non-human primate, mTOR, rapamycin, longevity, resilience

## Abstract

Traditional animal models have been used to make seminal discoveries in biomedical research including a better understanding of the biology of the aging process. However, translation of these findings from laboratory to clinical populations has likely been hindered due to fundamental biological and physiological differences between common laboratory animals and humans. Non-human primates (NHP) may serve as an effective bridge towards translation, and short-lived NHP like the common marmoset offer many advantages as models for aging research. Here, we address these advantages and discuss what is currently understood about the changes in physiology and pathology that occur with age in the marmoset. In addition, we discuss how aging research might best utilize this model resource, and outline an ongoing study to address whether pharmaceutical intervention can slow aging in the marmoset. With this manuscript, we clarify how common marmosets might assist researchers in geroscience as a potential model for pre-clinical translation.

## 1. Challenges of translation from laboratory animals (mostly rodents) to humans

Animal models have been a critical resource in the quest to evaluate physiological functional decline associated with aging and elucidate the cellular pathways and mechanisms associated with longevity. In general, these studies have focused most of their efforts on delineating the aging process using four primary laboratory organisms: yeast (*Saccharomyces cerevisiae*), roundworms (*Caenorhabditis elegans*), fruit flies (*Drosophila melanogaster*) and rodents including mice and rats (*Mus musculus* and *Rattus norvegicus*, respectively) [1]. These species have many practical advantages over other animal models (including humans) as models of aging including their relatively short lifespan, the ability to breed and maintain large numbers of animals in the laboratory, and the ability to control environmental exposure for each individual (or a population). In addition, over the last few decades it has been shown clearly that the relative ease of manipulating genes of interest; *i.e.*, generating organisms with specific genetic mutations to address mechanisms of phenotype including longevity, is a powerful tool for the study of aging. Using these models has been extremely fruitful for geroscience research and has driven discoveries of conserved biochemical pathways associated with longevity, and allowed the comparative evaluation of the effect of aging on metabolism and development, mitochondrial stress pathways, adenosine monophosphate-activated protein kinase, and insulin-like growth factor [2, 3]. In particular, the widespread use of mice, and in particular genetic mutant lines of mice, have driven exponential growth in our understanding of the particular mechanistic pathways on the mammalian aging process [4]. In terms of “translational” approaches to addressing aging and longevity in humans, mice and rats have served a central role in identification and elucidation of interventional and therapeutic treatments on aging and health-span. Most notably, the gold standard for extending lifespan in a model organism is the use of caloric restriction and indeed the seminal studies of caloric restriction have largely been performed using mice and rats [5-9]. In addition, many new drugs of geroscience interest have been tested in rodents and continue to reveal details regarding aging and disease progression, and have yielded novel findings about the anti-aging effect of intervention with pharmaceutical and nutraceutical compounds [10-17]. In many ways, interventional approaches utilizing pharmaceuticals seem to be the most “turnkey” for benefiting human health in terms of their relative ease of translation for clinical consideration.

However, there remain many concerns regarding using rodents as a model of human model of aging (in addition to disease, physiology, pathology, *etc.*) as a foundation for designing treatments and interventions to improve *human* health. Unfortunately, a litany of findings in rodent translational research have revealed that many of the drugs tested in rodents do not translate successfully to humans, either having few similar effects or having toxicity in humans [18]. Further, the development of inbred strains of mice has introduced the perpetuation of many traits that are a by-product of selection for breeding, rather than traits of interest. Many of these traits are undetected or unknown and may be detrimental to longevity studies or have unknown impact on the research of interest [19]. Lastly, there is a distinct dichotomy between rodents and humans in terms of the spectrum of diseases that naturally occur with old-age or otherwise. Particularly challenging are the wide-range of diseases and pathologies that plague aging humans that do not naturally exist in rodents (*e.g.*, neurodegenerative diseases including Alzheimer’s disease, cardiovascular disease, diabetes, and a host of others) due to differences between species in their basic physiology, the dramatic differences in their maximum ages, diet and environmental conditions or other unknown variables. Specifically because of such challenges, there has been significant investment in drawing from comparative biology to identify additional animal models that may serve as stronger bridges to clinical translation.

## 2. How non-human primates are useful (critical) to this process

Non-human primate (NHP) models are of particular interest for additional pre-clinical testing towards translation due to their close evolutionary history with humans. Old World monkeys and apes are the closest living relatives to humans and share a closer evolutionary history with humans than other mammals, and thus more similar genetic, biochemical, behavioral and phenotypic outcomes. Old world monkeys split from the ancestors of the human lineage around 25 million years ago, and apes, specifically chimpanzees, split as recently as six million years ago [20, 21]. In the limited studies that have examined the aging characteristics of captive chimpanzees, our closest living relatives, the age-related diseases in this species are described as similar to that of humans including the development of heart disease, cancer, and diabetes with advancing age [22-29]. However, chimpanzees are very long lived, with animals in captivity living up to 60 years, thus making them comparable in difficulty to working with human populations [21]. Additionally, both wild and captive populations of chimpanzees are now classified by the International Union for Conservation of Nature (IUCN) as endangered animalsand the National Institutes of Health (NIH) have effectively ceased funding for invasive research on chimpanzees. In part for these reasons, research using chimpanzees in the laboratory setting has significantly diminished in many countries including the U.S.

Other Old World monkeys, including rhesus macaques, have traditionally been of greater focus for biomedical translational research including aging studies [30-35]. Rhesus macaques have a maximum lifespan of approximately 40 years in captivity and their aging phenotypes have been well characterized [36, 37]. In addition, a means to monitor cognitive functional decline in this species has been developed and is hypothesized to model the similar decline in human cognition with age [32]. The rhesus macaque has also been used to test whether the benefits of caloric restriction on healthy aging translate to NHP species [30]. While there continues to be some discussion on the equivocal nature of caloric restrictions’ effect on NHP lifespan in two ongoing research groups, these studies have largely confirmed that calorie restriction reduces the prevalence of cardiovascular disease, type 2 diabetes, and neoplasias [38-45]. Due in part to the long lifespan of this species and the large time and financial commitment to these studies, it is unlikely that such in depth NHP lifespan studies will be able to be repeated.

In an attempt to reduce the heavy commitments required for aging NHP studies, several shorter-lived NHP species have been suggested for longevity research including the bush baby (*Galago senegalensis*), the grey mouse lemur (*Microcebus murinus*), and the common marmoset (*Callithrix jacchus*) [46, 47]. These species all have relatively shorter lifespans (for each, the maximum lifespan is ~15–20 years) compared to Old World monkeys and apes, and they offer a number of advantages due to their small size and rapid reproduction [46, 47]. Of note, it was recently reported that the lifespan of the grey mouse lemur could be extended by calorie restriction but that this intervention did not alter cognitive or motor function [48]. However, of these species, the common marmoset has recently emerged as a model for aging related research due to growing investigations on the changes in physiology, pathology and health that occur in this species [49-54].

## 3. Marmosets in aging research

**Fig. 1.**
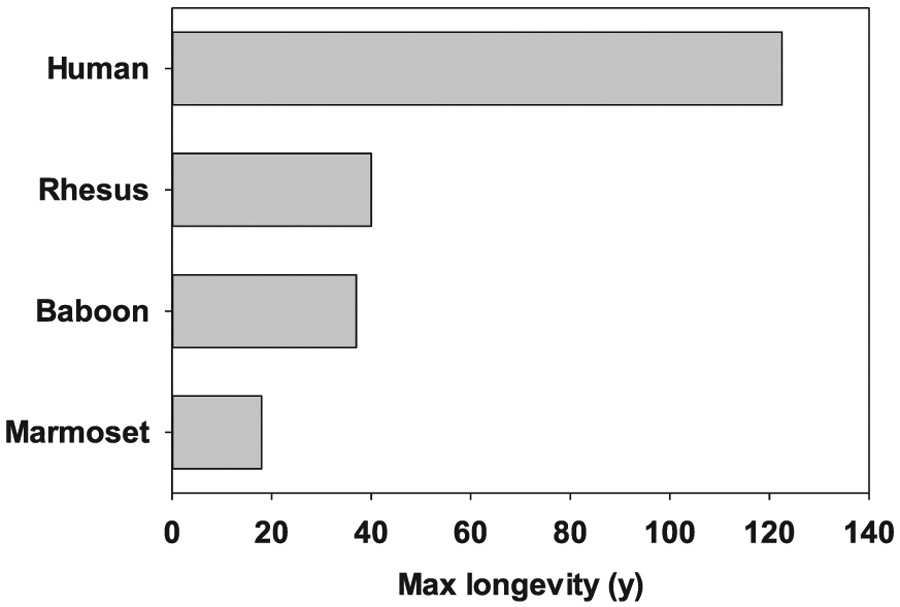
Maximum reported lifespans for four primary primate species used in aging research.

The common marmoset has many characteristics that make it attractive as a non-human primate model for aging research. As mentioned above, this species has a lifespan that is roughly half that of other NHP species commonly used in biomedical research (Fig. 1). While still quite long compared to rodents, this length of time is certainly more amenable to use within the career of a single researcher. Moreover, there is fiduciary benefit to the shorter lifespan (*i.e.*, half the amount of time paying per diems as other long-lived NHP). Along those lines, per diem costs tend to be significantly lower for marmosets than for other NHP, due in part to the smaller cage and space requirements. Their small size also has benefits in terms of pharmaceutical interventions (*i.e.*, less drug is required per animal) as well as in terms of staff safety. Marmosets are also the fastest reproducing anthropoid primate giving birth to fraternal twins on average twice per year [55]. This fast reproduction allows for relatively rapid growth of colonies for use in biomedical research. Further, these characteristics allowed for the development of an specific pathogen free (SPF) barrier maintained colony of marmosets for aging research which to date has displayed extended lifespan when compared to conventionally housed marmosets [56]. These features then support the notion that marmosets offer a unique NHP model with many advantages for studying the physiological changes associated with aging.

As part of the development of this species as an aging model, there is a growing understanding of the basic aging phenotypes of aging marmosets. For example, a number of skeletal changes associated with aging have been described in marmosets. Marmosets lack adult growth plates and display similar human age related structural changes in skeletal bone [57]. When treated with a bisphosphate therapy, trabecular number and volume increased in marmosets exhibiting age-related bone loss [57]. Further, marmoset bones undergo degeneration of structure associated with bone aging in such bones as the vertebrae [58].

With advancing age, marmosets have been reported to lose weight and show dramatic changes in body composition [54, 59]. These patterns are very similar to the types of changes in body weight and composition that occur in the latter half of normal lifespan in humans [60]. Interestingly, the maximum weight achieved by an individual marmoset is significantly associated with the likelihood of survival in older animals, with animals having a peak mass of less than 400 g having decreased survival after eight years of age [54]. Perhaps related to this, marmosets that exhibit increased rates of stretching behavior have a significantly higher 6-month mortality than animals do not display this behavior [50]. Rather than a beneficial behavior, this type of stretching is hypothesized to be a posture adopted due to discomfort in the marmoset. It is an atypical posture in which the marmosets hang for extended periods of time from their forelimbs rather than maintaining a more typical marmoset quadrupedal positioning, It is still unclear whether this can be attributed to a specific pathology but it would certainly be of further interest to identify how marmoset activity and ambulatory behavior are impacted by physiological declines associated with aging.

There has also been increasing interest in marmosets as a model to evaluate a number of age-related neurological diseases including Parkinson’s disease, Huntington’s disease, Alzheimer’s disease, stroke, multiple sclerosis and spinal cord injury. Marmosets exhibit decreased adult neurogenesis in the dentate gyrus that is evident before the animals would typically be classified as aged [61]. Ultrastructural examinations of the frontal cortex and hippocampus for animals more than 12 years of age found widespread accumulation of lipofuscin in the glial cells, perivascular macrophages and pericytes [62]. A recent study described an increase in α-synuclein aggregations in the olfactory bulb and hippocampus of aged marmosets [63]. β -amyloid deposition has been described in the brain of marmosets over the age of seven [64], and this data is often used as a criterion for defining marmosets as aged when they are eight years old. However, Ridley [65] did not detect *β* -amyloid in animals under the age of 10 in their colony and very little deposition in older animals. Marmoset brains have also been evaluated for the presence of amyloid beta markers and tau hyperphosphorylation [66] revealing diffuse amyloid plaques throughout the cortex of the aged marmosets, but not in the younger age groups. Conformational changes in tau were detected in all subjects in this study but these changes increased in frequency with aging. Dystrophic microglia were also significantly more likely to exhibit tau hyperphosphorylation than were active microglia in this study. However, the inconsistency between the presence of neurological markers at specific ages in the marmosets suggests that there may be environmental differences associated with the rate of aging between the colonies that have been examined.

As a potential translational model, there have also been numerous attempts to apply standard human clinical biomarkers of aging to marmosets. To date the only reported change in marmoset blood chemistry associated with age has been a significant decrease in albumin with advancing age [50]. Serum albumin decreases are of particular interest as they are a highly predictive of a risk of death in otherwise healthy aging humans [67]. In the largest study to date of age-related changes in marmoset blood chemistry, a cross-sectional analysis of 60 marmosets ranging in age from two to 13 years old identified 2500 metabolites [68, 69]. Connectivity between the metabolites was found to decrease with age and the abundance of several of the metabolites significantly declined with age [70]. With age, marmosets tend to display increased mean arterial pressure and diastolic pressure, but no age effect on the systolic pressure [71]. Finally, testosterone has been found to decrease in aging male marmosets, but they remain capable of reproduction, and no known phenotypic changes are associated with the decreased testosterone [55, 72-74].

**Fig. 2.**
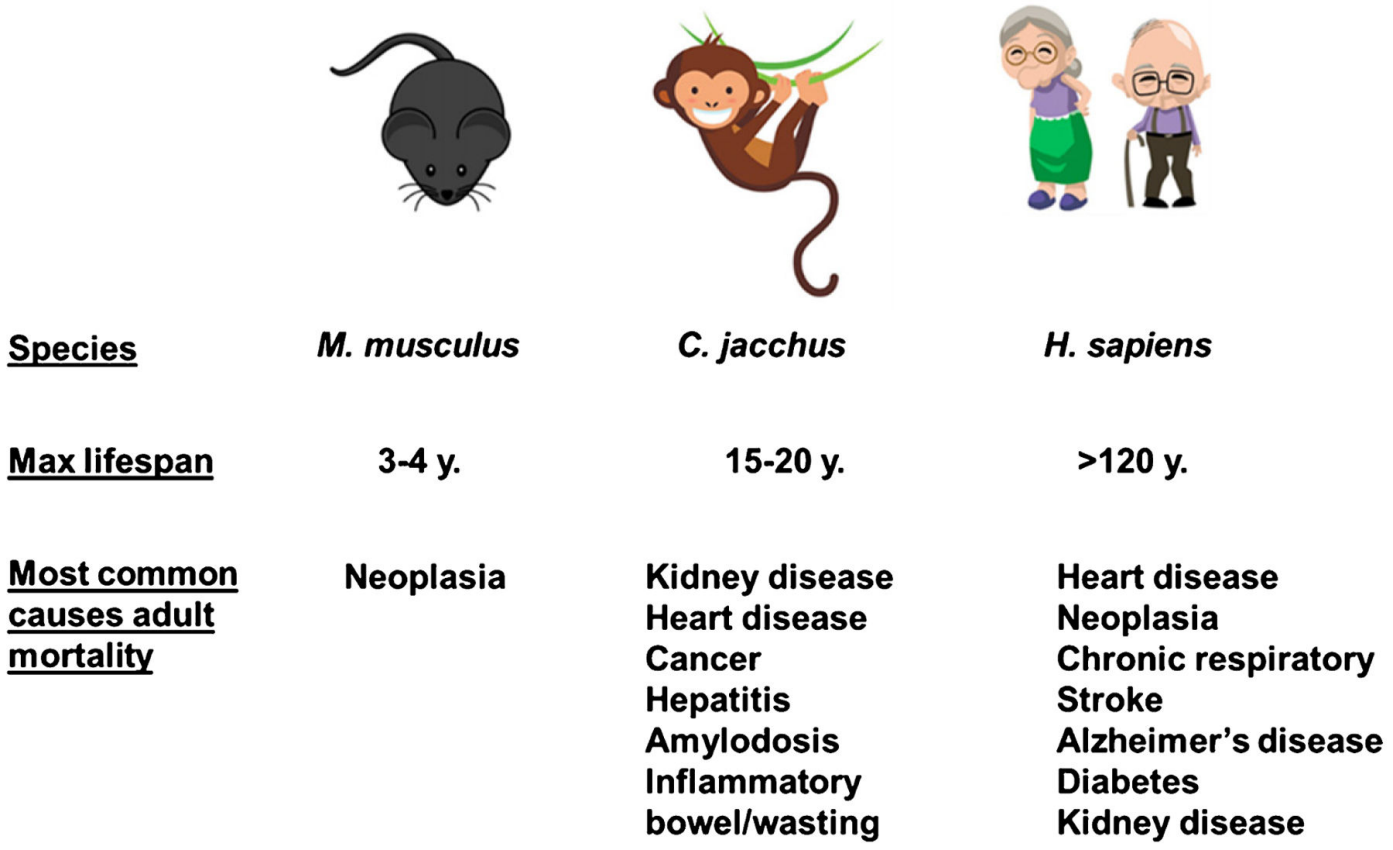
Common causes of death among laboratory mice, laboratory marmosets, and humans (clipart images from www.istockphotos.com).

An analysis of the pathologies associated with age in marmosets at the New England National Primate Center and the Southwest National Primate Research Center revealed dramatic shifts in the associated causes of death as animals aged in the colony [50, 54]. Deaths in young adults under the age of 6 years were most likely to be due to injury, inflammatory bowel disease (IBD) and infection. Both colonies reported rare occurrences of neoplasia and diabetes. In contrast, for marmosets over the age of 6 years the most likely causes of death were infection and IBD, with increasing rates of pathologies typically thought of as age related pathologies in humans and other model organisms including neoplasia, amyloidosis, diabetes, cardiac and renal failure. It must be noted that IBD is not a primary cause of mortality in humans and this reflects one of the challenges in using the marmoset as an aging model. On the other hand, the etiology of IBD in the marmoset has been difficult to ascertain and may be driven in part by susceptibility to infection in the community housing setting. Supporting this idea, we recently reported that marmosets maintained in SPF conditions showed no deaths associated with inflammatory gastrointestinal or infectious disease unlike marmosets maintained in a standard colony setting [56]. A brief summary and comparison of most likely causes of adult death among mice, marmosets, and humans are provided in Fig. 2.

While the extent of physiological and behavioral markers that have been evaluated for changes with aging is ever expanding, many things remain to be examined. For example, a common evaluation to assess frailty or health in geriatric patients is the use of walking speed and grip strength. Translating these relatively simple human assessments to marmosets has proven to be challenging. Other indicators of physiological and social resilience are also still lacking and need further refinement and development to determine their trajectories with age. Tools to assess cognitive health are not as varied and detailed in the marmoset as they are for human evaluations. Many of the cognitive tools used to evaluate Old World monkeys or apes are beyond the ability of the marmoset to be trained for or to complete, and interpretations of results can be controversial. Development of new cognitive tests and adaptations of human, monkey and mouse assessments are ongoing developments.

## 4. Scientific approaches to study aging in marmosets

It is now clear that longevity can be altered using three primary means in traditional laboratory animals: genes, diet, and pharmaceutical interventions [13, 75, 76]. These interventional methods are invaluable tools for studying the basic biology of aging and there is growing evidence that they can be translated to marmoset studies with some considerations.

While genetic modifications have been valuable tools in invertebrate and mouse models of aging, one of the biggest disadvantages historically for NHP biomedical studies has been the inability to produce genetically modified individuals to examine targeted areas of interest in ways that are either relatively cheap or easy (or hopefully both). There are a few reports of transgenic Old World monkeys that exist but the production of a single living offspring in these species is generally extraordinarily costly both financially and in terms of time commitment [77, 78]. Moreover, in terms of aging studies the phenotypic symptoms of the gene of interest may not appear until mid- to old-age which could be on the orders of decades. However, transgenic marmosets have existed for nearly a decade with the production of the lentiviral-induced GFP transgenic marmosets first reported in 2009 [79]. In this mutant monkey, the GFP transgene was distributed throughout the somatic cells in addition to a successful germ-line transmission that was verified within 2 years of the birth of the initial infants. Since this first report, a number of facilities have produced transgenic lines of marmosets using lentiviral transduction, CRISPR/Cas and Tet-on systems. A process that has a great deal of potential for the production of transgenic marmosets is somatic cell nuclear transfer (SCNT) because it may decrease the likelihood of producing mosaic individuals, but to date these attempts have not been successful [80]. Regarding aging research, many of the transgenic lines proposed and developed using marmosets have focused on neurologic disease and modeling of neurodegenerative disorders such as Alzheimer’s and Parkinson’s. Lentiviral transgenic induction of Tet-on human ataxin 3 genes has produced young marmosets with neurodegenerative disease phenotype [81, 82]. Recently, marmosets that express transgenic induction of intracellular calcium indicators have been developed in order to greatly advance the technology and imaging possibilities [83]. While the developing transgenic marmoset models is still certainly much more expensive and time consuming than developing similar rodent models, the ability to examine neurodegenerative disorders and cognitive deficit in a NHP will greatly advance our understanding of these processes in human aging. Further refinement of transgenic marmoset generation will also help expand the scientific focus of such models to other areas of aging research.

As mentioned above, calorie restriction has been one of most important tools used in aging research to understand the basic mechanisms of animal aging. Dietary interventions, including both dietary restriction and over-nutrition, have been adapted to marmoset studies to test the physiological outcomes of such interventions [84, 85]. However, these effects have not been evaluated in association with aging or age- related disease to date. Dietary restriction in marmosets has been limited to evaluations of the timing of caloric restriction during pregnancy and lactation to infant outcome [84]. While it is possible to restrict marmosets calorically, for some short studies such as measuring food intake or evaluation of daily food patterning, it is extremely challenging to use this intervention under normal housing conditions for this animal; *i.e.*, social housing. Marmosets are one of the few primates that has been found to actively share food with other family members and with infants [86]. This particular trait of marmosets makes it difficult to account for food taken from a hopper by an individual animal because even though they remove it from a dish, they are not necessarily eating the food by themselves. Single housing animals, as would typically be done in rodent calorie restriction, is not preferred in marmosets due to the social habits (and requirements) of this species. One potential approach to food restriction might be separating the individual of interest during feeding and then removing all food from the family housing prior to returning the animal. However, this technique is extremely labor intensive and it is likely that the entire group’s feeding patterns would be shifted in ways that would be challenging to document. Another possible approach would be to use time-restricted feeding or intermittent fasting, though the status of these approaches in comparison to “traditional” calorie restriction is still equivocal. Thus, there is still opportunity to develop appropriate caloric restriction paradigms in the marmoset for future testing.

Pharmaceutical interventions are by far the most easily translated to marmoset studies of aging and age-related disease. The marmoset is already a valuable NHP model for evaluating preclinical drug pharmacokinetics, teratology and toxicity in part due to its small size and thus need for smaller doses of drugs compared to other NHP [87-89]. Their metabolic profile and primate physiology allows drugs to be tested in the same delivery modality as will be used in human dosing, making translation to clinical trials smoother. Marmosets have been key to the development of drugs for use in neuroscience, immunology and infectious disease [89]. Drug development and testing for multiple sclerosis, Parkinson’s, stroke, spinal injury, amyloidosis, hepatitis, encephalitis, viral hemorrhagic fevers and bone disease have all relied on marmosets [89]. Thus, an easy case can be made for using this particular approach as the “low-hanging fruit” for refinement of the marmoset as a valuable NHP for studying the basic biology of aging. In this regard, we have put together an aging cohort of marmosets to test the very idea of whether drug intervention can delay the aging process in this NHP.

## 5. Designing a study to address whether rapamycin slows aging in marmosets

The work of the Interventions Testing Program and others has identified rapamycin as one of the most consistent (so far) pharmaceutical interventions capable of extending lifespan and improving health in mice [13-15, 17, 90]. Rapamycin is an inhibitor of mTOR (mechanistic target of rapamycin) signaling and this compound, along with some of its analogues, have been shown to delay (if not rejuvenate) the physiological dysfunction and pathologies associated with aging across multiple organs in the aging mouse [90-96]. Collectively, these outcomes have provided the first proof of principle that longevity in mammals can be extended, if not slowed outright, by administration of a pharmaceutical agent. As described above, there are significant challenges in applying the results of intervention testing in rodents towards the benefit of human health. There is some evidence that rapamycin or its orthologs might provide some benefit to generally healthy older human populations with limited side-effects [97,98]. While clinical based trial may reveal additional benefit, they are unlikely in the near future to be capable of determining whether this intervention increases longevity or slows aging among humans directly. As discussed here, there then is potentially tremendous benefit in addressing whether interventions that extend longevity in mice, such as rapamycin, similarly extend lifespan in aNHP species.

To this end, we have enrolled a cohort of middle-aged marmosets into a long-term study to test the effect of rapamycin on longevity and healthy aging in this species. During the development of this project, we previously characterized the pharmacokinetics of daily oral treatment with rapamycin encapsulated in a slow-release enteric coating (as used in the Intervention Testing Program’s mouse studies). We reported that marmosets treated with an oral dose of 1.0 mg/kg body weight/day rapamycin showed circulating trough rapamycin concentrations similar to those reported in mouse lifespan studies as well as similar to the reported therapeutic ranges for humans given rapamycin as chemotherapy [99]. Moreover, we showed this dose of rapamycin was sufficient to inhibit mTOR signaling *in vivo* both in the short (weeks) and long (14 months) term. In this pilot study, we also reported little to no signs of intolerability among animals treated with rapamycin (total 7 animals of mixed sexes) and no additional veterinary interventions were required of animals who had taken daily treatment with this drug for approximately 14 months.

Our results from this pilot study in large part confirmed that we could deliver rapamycin to marmosets in such a way to at least achieve similar blood concentrations (and *in vivo* mTOR inhibition) similar to that reported in mouse longevity studies using this drug. While this suggests the basic biochemical properties of this drug intervention can be translated between these species, other results from our pilot study support the notion that physiological outcomes of such an intervention may differ between species. For example, one of the relatively consistent ancillary effects of rapamycin treatment in mice has been development of glucose intolerance, likely due to an inhibitory effect of rapamycin on mTORC2 signaling in the liver [15, 100-102]. However, in marmosets treated with rapamycin we found no evidence of glucose intolerance measured by oral glucose tolerance tests or indices of insulin resistance [103]. It is unclear exactly why we found these discrepancies between species, though one possibility might be basic anatomical differences between mice and NHP. For example, the liver, which has been reported to be at least partially responsible for rapamycin-mediated glucose intolerance, makes up a greater contribution of the total mass of mice in comparison to marmosets. In this regard, marmoset relative liver mass is much more similar to humans than to mice. While this provides no assurance that results from marmosets will directly translate to humans, it does hint at the potential value of utilizing a model species with basic biological characteristics similar to humans in biomedical research.

In a similar vein, there has been speculation that at least part of the beneficial effects of rapamycin on longevity are mediated through mTOR’s effects on protein homeostasis (or proteostasis). While the relationships between mTOR and autophagy and mTOR and protein translation are well-understood, there is also growing evidence that mTOR may regulate proteostasis through other pathways such as ubiquitin-proteosome and protein chaperone activation. Indeed, previous reports have suggested that chronic rapamycin treatment can stimulate autophagy, induce proteosome activity and increase expression of protein chaperones [104-107]. In marmosets treated with rapamycin, we found evidence for mild stimulation of autophagy in some, though not all, tissues compared to control animals though little to no evidence that other protein degradation pathways, including proteasome and protein chaperones, were affected [108]. These data then suggest that if rapamycin has an effect on healthy aging in the marmoset, at least some of this effect might be contributed to activation of autophagy and provides a mechanistic target of action for further study.

The goal of our ongoing marmoset study is to directly address the question of whether intervention with rapamycin will benefit longevity or healthy aging in this species. As mentioned above, we have recruited and enrolled a cohort of middle-aged marmosets into a long-term study to test this question. We have designed this experiment starting with older animals (approximately 5–7 years of age in this species) for three main reasons. 1) This provides a reasonable chance to see effects on longevity in this species over the next 5–10 years. 2) This is similar to the original Interventions Testing Program report on rapamycin which started in middle-aged mice and showed a beneficial effect of this drug on longevity [13]. 3) It is reasonable to assume that translation of aging interventions in humans might target those individuals that have reached a period considered later in life [97]. Our cohort is of mixed sex with roughly equivalent numbers of male and female animals. We are treating half of the cohort with rapamycin administered daily at dose of 1 mg/kg body weight which we have shown is comparable to doses known to extend mouse lifespan [99], the other half of this cohort is control (*i.e.*, treated daily with the agent used to encapsulate rapamycin only). We are tracking physiological and behavioral phenotypes as well as health outcomes and longevity over the next several years.

While our primary goal is to assess effects of this intervention on longevity in the marmoset, we also have the opportunity to examine the effects of rapamycin on “healthy aging” or the progression of age-related physiological decline. In mice, rapamycin has been suggested to delay this progression (or even reverse age-related loss) across several organ systems with cardiac [91, 93], hematopoietic stem cells [96], the immune system [94], and periodontal bone [109] benefits among the many reported positive outcomes. In our cohort of aging marmosets, we are testing the effect of both advancing age and rapamycin intervention across multiple minimally-invasive assessments of animal health, frailty and resilience. For example, changes in blood chemistry, complete blood counts, blood lipids, etc. are being tested through regular blood draws similar to what would be performed during routine physical assessments in a clinical setting. In addition, we have defined scheduled tests of glucose metabolism, cardiovascular function, musculoskeletal function and inflammatory processes outlined for repeated testing throughout the remaining lifetime of this cohort. These repeated longitudinal tests also give us an opportunity to address long-term administration of rapamycin to a relatively healthy population has similar potential for side-effects as in clinical use to treat specific disease conditions. For example, the most commonly cited potential side effects of this drug are metabolic dysfunction (including new-onset type 2 diabetes and dyslipidemia) and immunological suppression. As mentioned above, we have found so far no evidence to suggest rapamycin is impairing glucose metabolism at least through approximately 1 year of treatment and no evidence for dyslipidemia [51]. We continue to monitor these outcomes in our long-term treatment cohort. While our design does not allow us to directly test changes in immune response with age and rapamycin in the marmoset, we do monitor changes in blood cell count (including subsets of leukocytes) during our semi-annual health checkups. Again, to date we have found little evidence that chronic rapamycin significantly alters these parameters. Functional tests of immune cell function *in vivo* would at least tangentially address whether rapamycin inherently alters the ability of the immune cells to address challenge.

At death, animals will undergo full pathological assessment as well as to tissue collection to assess the effect of chronic intervention with rapamycin on multiple markers that represent the “pillars of aging” [110]. Thus, over the course of this study we should develop a complete picture of not only the broad effects of aging on the common marmoset but also whether pharmaceutical interventions known to extend lifespan in mice have similar effects in a NHP species.

## 6. Conclusions

Marmosets offer a unique non-human primate model in which to evaluate both cross-sectional and longitudinal effects of aging on measures of longevity and healthspan. While areas of interest remain for which there are currently no assessment tools for use in the marmoset, recent developments have rapidly expanded tools that are available. Marmosets have significant advantages including relatively short life span and ease of handling that make them ideal for this type of work. We look forward to the continued expansion of tools and knowledge that are becoming available for the marmosets as models for aging research.
